# Suicide by homeless patients in England and Wales: national clinical survey

**DOI:** 10.1192/bjo.2021.2

**Published:** 2021-03-12

**Authors:** Paul Culatto, Lana Bojanić, Louis Appleby, Pauline Turnbull

**Affiliations:** Greater Manchester Mental Health NHS Foundation Trust, UK; National Confidential Inquiry into Suicide and Safety in Mental Health, Faculty of Biology, Medicine and Health, The University of Manchester, UK; Greater Manchester Mental Health NHS Foundation Trust, UK; and National Confidential Inquiry into Suicide and Safety in Mental Health, Faculty of Biology, Medicine and Health, The University of Manchester, UK; National Confidential Inquiry into Suicide and Safety in Mental Health, Faculty of Biology, Medicine and Health, The University of Manchester, UK

**Keywords:** Suicide, homeless, in-patient treatment, discharge, substance misuse

## Abstract

**Background:**

Homelessness in England and Wales is on the rise together with the mortality rate among homeless people. Many homeless people have a mental illness, which is a risk factor for suicide.

**Aims:**

This study used data from the National Confidential Inquiry into Suicide and Safety in Mental Health to examine demographic and clinical characteristics of homeless people who died by suicide and were in recent contact with mental health services.

**Method:**

We have compared 514 patients (2% of the total sample) who died by suicide and who were reported as being homeless or having no fixed abode by their clinicians with patients in stable accommodation between 2000 and 2016 to identify differences in sociodemographic characteristics and clinical care.

**Results:**

Our analysis suggests that homeless patients who died by suicide had more acute (alcohol: 47% *v*. 25%, *P* < 0.01, drug: 39% *v*. 15%, *P* < 0.01) and chronic (alcohol: 72% *v*. 44%, *P* > 0.01, drug: 64% *v*. 31%) substance misuse issues than patients in stable accommodation. Homeless patients were also more likely to die as in-patients (21% *v*. 10%, *P* < 0.01) or within 3 months of discharge (32% *v*. 19%, *P* < 0.01).

**Conclusions:**

Homeless patients who died by suicide more often had known risk factors for suicide than patients in stable accommodation. As a result of the higher percentages of post-discharge and in-patient suicides in homeless patients as well as the high prevalence of substance misuse, this study recommends closer integration of services as well as awareness of risks during in-patient admission and in the weeks immediately after discharge.

Homelessness in England and Wales is rising; the number of people sleeping rough in England increased more than 2.5 times from 2010 to 2017^1^ and 1.5 times in Wales from 2015 to 2018.^2^ At the same time, figures released by the Office for National Statistics (ONS) estimate that there has been a 51% increase in deaths of homeless people from 2013 to 2018 in England and Wales.^3^ High mortality rates among homeless people are common in high-income countries, with especially high excess mortality for suicide in users of homeless shelters compared with the general population.^4^ Homeless people who die by suicide are more likely to be male, young, unmarried, unemployed, to have had at least one physical illness or other stressful life event prior to death, and to have drug and alcohol misuse.^5–7^

Additionally, people who are homeless have a higher proportion of mental disorders than people with stable accommodation, particularly psychotic illness, personality disorders and substance misuse.^8–10^ A German study^11^ found a 3.5 times increase of primary mental disorder in homeless people compared with the general population, with alcohol and drug dependency accounting for the majority of these diagnoses. Among the homeless population using shelters in Denmark, the proportion of diagnosed psychiatric disorders is estimated as 62% for men and 58% for women.^4^

Research from the National Confidential Inquiry into Suicide and Safety in Mental Health (NCISH) into suicide by mental health patients who were homeless found they were more likely to have drug and alcohol problems, recent suicidal ideation and behaviour, and a history of disengagement from services compared with non-homeless patients who died by suicide.^12^ As that study was undertaken in 2006, an updated review of characteristics and clinical care received by homeless people prior to suicide is needed, in the context of increasing numbers of rough sleepers and their specific mental health needs. Our study compares patients who died by suicide in England and Wales and were homeless at the time of death with those in stable accommodation.

## Definition of homelessness

The term homelessness goes beyond describing people sleeping on the streets with no roof over their head, which is often described more specifically as ‘rough sleeping’. It includes two other categories of homelessness:^13^ those in temporary accommodation, including hostels and bed and breakfasts, and the ‘hidden homeless’, who have no home but find somewhere temporary to live for example ‘sofa-surfing’ or in squats. Defining homelessness is not straightforward; there are slight variations in the definitions of homelessness even between the UK's devolved nations.^13,14^

## Method

This study was conducted within the NCISH. NCISH collects data on all deaths by suicide by people in England and Wales who were in contact with mental health services in the 12 months prior to suicide (further referred to as ‘patients’). The method of the NCISH data collection is fully described elsewhere.^15^ In short, data collection starts with the identification of all people who died by suicide in the UK. Then, information about whether the deceased had been in contact with mental health services in the 12 months before death is obtained from the National Health Service (NHS) trusts in the deceased's district of residence. Lastly, clinical data is obtained via questionnaires completed by the patient's supervising clinician. Questionnaires collect information regarding the patient's demographic characteristics, psychosocial history, details of suicide, treatment and adherence, last contact with services prior to death, and the clinicians’ view on possible suicide prevention.

The NCISH achieves a questionnaire response rate of >95%. NCISH has research ethics approval from the North West Research Ethical Committee and approval under Section 251 of the NHS Act 2006 (originally Section 60 of the Health and Social Care Act 2001). This means that the person responsible for the information, in this case clinicians, can disclose confidential patient information without informed consent in the UK.

## Statistical analysis

Descriptive statistics are presented as valid percentages with 95% CI for homeless and non-homeless patients. Differences between those two groups have been assessed with the χ^2^ tests. Since age was the only continuous variable, it is reported using median and interquartile range and the difference in the age between the two groups was assessed using the Kruskal–Wallis test. Significance was reported using *P*-values, and results with *P*-values less than 0.05 were considered significant. All analyses were carried out using Stata 15 software.^16^

## Results

From the year 2000 to 2016 there were 22 403 deaths by suicide where the person had been in contact with services within the past 12 months (referred to throughout as patients). The sample was divided into homeless and non-homeless patients based on the accommodation status reported on the questionnaire. There were 514 people reported as ‘homeless/ of no fixed abode’, 121 were living in a long-term bed and breakfast, 783 living in a supervised or unsupervised hostel or local authority accommodation, <3 living in a secure children's home/ secure training centre, 19 295 living in a house or flat, 143 living in a prison/young offenders institution, 138 living in a nursing or care home, and 515 in other accommodation (not otherwise specified).

For the purposes of this study, we have selected the accommodation category homeless/no fixed abode as our ‘homeless’ group (*n* = 514), this corresponds to the definitions of rough sleeping and the hidden homeless quoted in other literature.^13^ It is worth noting that some of the aforementioned 515 patients whose accommodation was listed as ‘other’ might also have been homeless; we were, however, unable to identify them from the available data. The numbers for the ‘non-homeless’ group are the sum of all accommodation categories excluding homeless/no fixed abode (20 997). The discrepancy between this number and the total is because of missing accommodation information in questionnaires; this information was missing for 892 (4%) patients.

### Demographic characteristics and antecedents

**Fig. 1:**
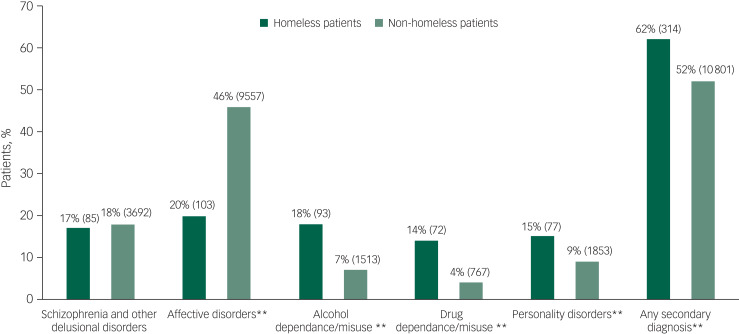
Comparison of primary diagnosis between homeless and non-homeless patients who died by suicide. All percentages are valid percentages (**P < 0.01).

**Fig. 2:**
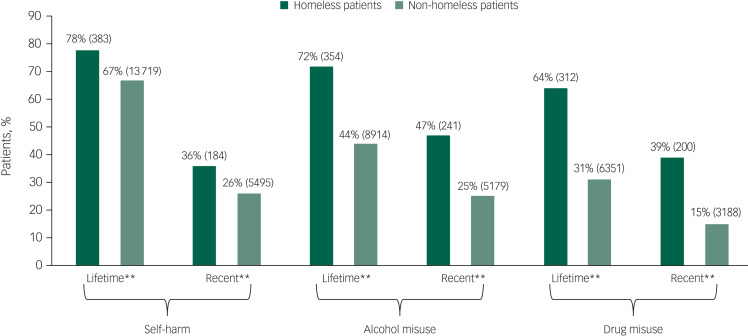
Comparison of lifetime and recent (<3 months before death) behavioural characteristics between homeless and non-homeless patients who died by suicide. All percentages are valid percentages (**P < 0.01).

Homeless patients who died by suicide were younger, more likely to be male and unemployed compared with their non-homeless counterparts. Further, fewer homeless patients died by self-poisoning (Table 1). They were significantly more likely to have a diagnosis of alcohol or drug dependence, personality disorder or a secondary diagnosis (Fig. 1), and less likely to have affective disorders. Homeless patients had a significantly higher experience of self-harm, alcohol and drug misuse, both in their lifetime and within the last 3 months (Fig. 2).

**Table 1:** Comparison of demographic characteristics and methods of suicide between homeless and non-homeless patients who died by suicide. All percentages are valid percentages.

Characteristic	Homeless patients	Non-homeless patients	*P*^a^
Total, *n* (%)	514 (2)	20 997 (98)	–
Demographic characteristics			
Age, median (minimum–maximum)	37 (28–45)	46 (35–57)	< 0.01
Gender, male: *n* (%)	431 (84)	13 747 (66)	< 0.01
Ethnic minority, *n* (%) 95% CI	40 (8) 5.7–10.7	1512 (7) 7.0–7.7	–
Unemployed, *n* (%) 95% CI	404 (81) 77.2–84.3	8534 (42) 41.2–42.6	< 0.01
Patient was a former member of the Armed Forces, *n* (%) 95% CI	4 (2) 0.6–5.2	187 (3) 2.3–3.0	–
Method of suicide, *n* (%) 95% CI			
Hanging/strangulation	235 (46) 41.4–50.2	8750 (42) 41.1–42.4	–
Self-poisoning	99 (19) 16.0–23.0	5290 (25) 24.7–25.9	< 0.01
Carbon monoxide poisoning	19 (4) 2.2–5.7	569 (3) 2.5–3.0	–
Jumping/multiple injuries	88 (17) 14.0–20.7	3182 (15) 14.7–15.7	–
Drowning	28 (6) 3.7–7.8	1192 (6) 5.4–6.0	–
Other	44 (9) 6.3–11.3	1931 (9) 8.8–9.6	–

a. Only significant *P*-values are shown.

### Suicide methods

Homeless patients who died by suicide were less likely to die by self-poisoning compared with their non-homeless counterparts (Table 1). However, homeless patients who died by self-poisoning were more likely to overdose on opiates (50% *v*. 22%, *P* < 0.01).

### Clinical care characteristics

**Fig. 3:**
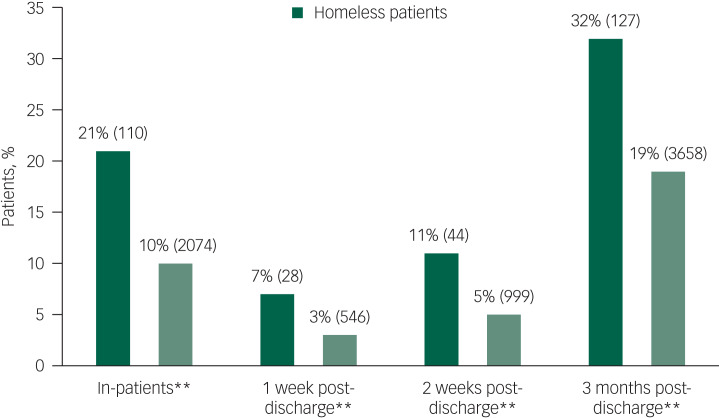
Comparison of in-patient and recent post-discharge suicides between homeless and non-homeless patients who died by suicide. All percentages are valid percentages (*P < 0.05, **P < 0.01).

A total of 21% of homeless patients died while on psychiatric in-patient wards compared with 10% of non-homeless patients (Table 2). Of the homeless people who died in in-patient care, 43% died while being off the ward without staff agreement, including with staff agreement but failing to return. This was significantly more than non-homeless in-patients (30%, *P* < 0.05). In the post-discharge period, there were significantly more deaths by suicide in the first week, 2 weeks and 3 months in the homeless population compared with the non-homeless population that died within this time frame of 3 months (Fig. 3).

**Table 2:** Comparison of clinical characteristics between homeless and non-homeless patients who died by suicide. All percentages are valid percentages.

	*n* (%) 95% CI		
Characteristic	Homeless patients	Non-homeless patients	*P*-value^a^
Total, *n* (%)	514 (2)	20 997 (98)	–
In-patient care			
In-patient at time of death	110 (21) 17.9–25.2	2074 (10) 9.5–10.3	< 0.01
Patient discharged to housing, financial and/or employment problems	10 (83) 51.6–97.9	92 (20) 16.0–23.4	< 0.01
Under the following service at the time of death			
Crisis team	36 (8) 5.9–11.4	2468 (13) 12.4–13.3	< 0.01
Seen in emergency department for self-harm 3 months prior to suicide	89 (26) 21.1–30.6	2557 (15) 14.3–15.4	< 0.01
Assertive outreach	11 (3) 1.3–4.5	570 (3) 2.8–3.2	–
Drug services	27 (18) 12.2–25.1	440 (7) 6.0–7.2	< 0.01
Alcohol services	33 (22) 15.9–29.9	469 (7) 6.5–7.7	< 0.01

a. Only significant *P*-values are shown.

Homeless patients were less likely to be under the crisis resolution home treatment team providing intensive home treatments for acute mental health crisis (8% *v*. 13%). They were also more likely to have been seen in the emergency department for self-harm in the 3 months prior to their death and to be under drug or alcohol services (Fig. 2, Table 2).

### Last contact

Unsurprisingly, homeless patients were less likely than patients with stable accommodation to have had their last contact with services at home (5% *v*. 27%) They were more likely to have been seen in an emergency department (8% *v*. 3%), psychiatric ward (27% *v*. 11%) and in other settings (10% *v*. 3%). The other settings in question were most commonly: addiction services, with friends or family, and police. When clinicians were asked what would have helped prevent the suicide, better crisis facilities (15% *v*. 9%) and availability of dual diagnosis services (21% *v*. 10%) were more commonly suggested for homeless patients than patients in stable accommodation.

### Confounding effects of age, gender and calendar year

Potential confounding effects of age, gender and calendar year were explored via logistic regression analysis with homelessness as a criterion variable. Logistic regression analysis was carried out in forwards stepwise fashion (see Supplementary Appendix 1 and Supplementary Table 1 available at https://doi.org/10.1192/bjo.2021.2); model A has demographic and clinical characteristics as well as method of suicide entered as predictors in one step. Models B, C and D contain age, gender and calendar year of death, respectively, as predictors to explore their potential confounding effects. Even though model fit improved by adding age, gender and calendar year, overall significance of predictors from model A did not change.

## Discussion

To the best of our knowledge, this is the first study to examine demographic and clinical characteristics of homeless mental health patients who died by suicide in the England and Wales since 2006.^12^ Additionally, this study benefits not only from inclusion of in-depth clinical data, but also clinicians’ perspectives on suicide prevention. By comparing these characteristics with those of patients in stable accommodation, we have identified and highlighted differences that can help with the provision of clinical care for homeless patients, such as differences in acute and chronic substance misuse issues, timing of suicide and ways of accessing services.

### Diagnosis

Previous studies have clearly demonstrated the effect of substance misuse on increasing suicide risk.^3,4,10^ Our study is consistent with these results. We found that homeless patients who died by suicide were more likely than those in stable accommodation to have a primary diagnosis of substance misuse, previous history of substance misuse, and to have been under drug and alcohol services at the time of their death. Clinicians also stated that improved access to a dual diagnosis service could have helped to prevent suicide in this group. Higher prevalence of substance misuse is also reflected in higher prevalence of self-poisoning deaths by opiates overdose in homeless patients, despite overall fewer deaths by self-poisoning; this is consistent with previous research.^7,12^

The most recent ONS report on deaths of homeless people at the time of publication mentions substance misuse as an important factor in their increased mortality.^3^ Since substance misuse has been found to be associated with both suicidal ideation and attempts in homeless people,^17^ it is important to involve homeless people in substance misuse treatment to reduce the risk of suicidal behaviours. Comorbid mental disorder and substance misuse often precedes suicide, and so close integration of substance misuse services with mental health services is an important step in risk prevention for this group.^17^

It is important to note comparable prevalence of schizophrenia and other delusional disorders in homeless and non-homeless patients who died by suicide in our sample; this is contrasting to the findings of a recent meta-analysis that has found significantly higher prevalence of schizophrenia and other delusional disorders in homeless people.^19^ This difference was not as prominent when looking at data solely from high- and middle-income countries, which may explain our finding to some extent. Additionally, it is possible that this difference stems from the fact that the data for this study is only from people in recent contact with a mental health service who died by suicide. Finally, our sample comprised mental health patients who were more likely to be diagnosed using diagnostic criteria and instruments, rather than screening instruments, which could have contributed to lower overall prevalence of delusional disorders.^19^

Homeless patients who died by suicide had significantly lower prevalence of depression as the primary diagnosis compared with the non-homeless patients. Even though one would expect depression to be higher in this population given the stressors that being homeless entail, previous research also found the rates of depression in the homeless population to be lower than expected.^9^ Fazel and colleagues hypothesised that this discrepancy may be because of suicide in homeless populations being mediated through other risk factors, such as substance misuse, rather than through depression.^9^ This hypothesis is supported in our study by the significantly higher prevalence of a diagnosis of drug and alcohol misuse and personality disorder which, to some extent, could also be masking an underlying depression.

### In-patient and post-discharge suicide

Homeless patients are more likely to die by suicide during an in-patient admission and the post-discharge period than non-homeless patients, as can be seen in Fig. 3. Increased risk of death by suicide in those that have had previous contact with mental health services has been recognised both in the general population^20^ and homeless people.^10^ We found that the percentage of homeless people who died by suicide under psychiatric in-patient care was double that of non-homeless in-patients.

Even though the total number of in-patient suicides is falling,^21^ this may not be the case in this particular patient subgroup. It is also important to note that over 40% of homeless in-patients died while being off the ward without staff agreement. Previous research on in-patients who have died by suicide off the ward without staff agreement – so-called absconders – also noted higher rates of homeless patients dying under these circumstances, and recommended tighter observation of ward exits, individually tailored patient observation levels and improvement of the ward environment as prevention measures against absconding.^22^

In addition to this, homeless patients’ percentages of post-discharge suicides are higher at all post-discharge time points than their non-homeless counterparts that have also died within the said time frame. It is noted that homeless patients are more likely to be discharged from in-patient care into housing, financial, and/or employment problems (i.e. being unemployed). Even though these areas are part of the standard risk assessment carried out prior to discharge from mental health services, it is probable that clinicians face significant limitations in addressing difficulties in these areas, thus being unable to reduce the risks. For patients, facing these problems again may contribute to the increased number of post-discharge suicides in the homeless patient group.

### Contact with services

Homeless patients were more likely to be under drug and alcohol services or have been seen in the emergency department following self-harm than non-homeless patients. Clinicians felt that improved access to crisis facilities could have made the suicide significantly less likely at the time for homeless patients; this is consistent with the finding that homeless patient were less likely to be under a crisis team at the time of their death. Crisis teams often rely on good engagement from the patient although this may be difficult for someone who is homeless. This can also be the case for patients under community mental health teams, who may lack the resources to reach people who are homeless. In the UK, where the provision of homeless mental health services is variable across the countries with funding streams often being uncertain, the roles of community mental health teams in patient care can vary from assessment to a full provision of care.^23^

Additionally, a recent NCISH report states that the majority of homeless patients have been registered with a general practitioner in the year before their death by suicide.^21^ Even though homeless mental health patients may be seen as a hard to reach group, there is some evidence to suggest that they are in contact with services, although it is possible that their pattern of service use is different to that of non-homeless patients who died by suicide, namely, by coming to emergency departments more. It may be that because of sporadic contact or the isolated working of services crucial opportunities for suicide prevention have been missed. This highlights the necessity to develop services that cater to different patterns of contact by homeless patients and that can consider and address some of the relational difficulties that can interfere with meaningful engagement.

### Limitations/methodological issues

As this is an exploratory, uncontrolled retrospective study, we cannot make any causal inferences, and therefore the descriptive findings should be interpreted with caution. There are some previously established limitations of the NCISH methodology, such as potential bias of the clinicians providing information.^24^ Even though we have minimised the possibility of missing certain suicide deaths by including those patients whose deaths were classified as open verdicts, it is possible that we missed some of the homeless patients that belong in the so-called ‘hidden homeless’ category such as those living in hostels and bed and breakfast. Also, many of the significant findings could be related to the state of homelessness patients in general, rather than being specific to homeless patients who die by suicide. Conversely, findings from this study cannot be generalised to the general population of homeless people, in which undiagnosed mental illness is often a problem. It is important to note that most homeless people do not contact mental health services unless this is actively sought by homeless treatment teams specialising in their care.^25^ The comparison shows instead whether homeless patients who died by suicide have a different pattern of risk from non-homeless patients and serves to highlight the suicide prevention measures that could have most effect for these individuals. As diagnoses were made and reported by the treating clinician, we cannot be sure which categorisation or diagnostic tools were used. Finally, we had no information about the length of homelessness for the deceased patients.

### Clinical implications

There is already some work being done to help homeless people with mental health problems including the use of psychologically informed environments, an approach that helps service providers remodel services in order to better address psychological issues that homeless people face.^26^ Psychologically informed environments has been used in the creation of specialised homeless mental health teams with some positive initial results.^23^ Further, the publication of standards for commissioners and service providers for people with complex problems^27^ has helped commissioning of services for homeless people. These standards aim to support homeless people by improving access, engagement and liaising between services. Homeless people attending emergency departments while in crisis or being admitted to a physical health hospital may provide an opportunity for engagement with services and may require a proactive approach by mental health services. The Faculty for Homeless and Inclusion Health have created specialist teams of general practitioners that provide in-reach in physical health hospitals, a model that could be implemented in mental health hospitals.^28^

Providing appropriate help for mental health patients with substance misuse has become more challenging since the NHS reforms that have distanced substance misuse provision from mental health provision.^29^ This poses an additional risk as now patients with comorbidities need to access two separate services, one for mental health and one for substance misuse issues. This can be especially challenging for people without stable accommodation in an out-patient setting and can mean lack of substance misuse therapy in an in-patient setting. Services should therefore be well connected with each other and aware of the nature of engagement with services that homeless patients can have. Additionally, as evident from the high numbers of in-patient and post-discharge suicides among homeless patients, in-patient units should be aware of the increased risks of suicide during admissions and in the post-discharge period, and provide appropriate discharge planning and follow-up for homeless patients.

### Research implications

Additional research is needed into homeless patients’ experiences of hospital admissions and discharge. Considering the high rates of in-patient suicides for this group, particularly when off ward without agreed leave, a deeper examination of their experiences during hospital admissions is warranted. Qualitative investigation with homeless patients and mental health staff, either through interviews or focus groups, might help to provide much needed insight into the particular difficulties they face during their hospital admission.

## Data Availability

The data that support the findings of this study are available on request from the corresponding author, L.B. The data are not publicly available because of their highly sensitive nature.

## Supplementary material

For supplementary material accompanying this paper visit http://dx.doi.org/10.1192/bjo.2021.2. click here to view supplementary material

## References

[1] Ministry of Housing, Communities and Local Government. Rough Sleeping Statistics Autumn 2018. Ministry of Housing, Communities and Local Government, 2019.

[2] Statistics for Wales. National Rough Sleeper Count, November 2018. Welsh Government, 2019 (https://gov.wales/sites/default/files/statistics-and-research/2019-02/national-rough-sleeper-count-november-2018_1.pdf).

[3] Office for National Statistics. Deaths of Homeless People in England and Wales: 2018. Office for National Statistics, 2019 (https://www.ons.gov.uk/peoplepopulationandcommunity/birthsdeathsandmarriages/deaths/bulletins/deathsofhomelesspeopleinenglandandwales/2018).

[4] Nielsen SF, Hjorthøj CR, Erlangsen A, Nordentoft M. Psychiatric disorders and mortality among people in homeless shelters in Denmark: a nationwide register-based cohort study. Lancet 2011; 377: 2205–14. 21676456 10.1016/S0140-6736(11)60747-2

[5] Arnautovska U, Sveticic J, De Leo D. What differentiates homeless persons who died by suicide from other suicides in Australia? A comparative analysis using a unique mortality register. Soc Psychiatry Psychiatr Epidemiol 2014; 49: 583–9. 24100916 10.1007/s00127-013-0774-z

[6] Barak Y, Cohen A, Aizenberg D. Suicide among the homeless: a 9-year case-series analysis. Crisis 2004; 25: 51–3. 15387210 10.1027/0227-5910.25.2.51

[7] Sinyor M, Kozloff N, Reis C, Schaffer A. An observational study of suicide death in homeless and precariously housed people in Toronto. Can J Psychiatry 2017; 62: 501–5. 28525964 10.1177/0706743717705354 PMC5528989

[8] Rees S. Mental Ill Health in the Adult Single Homeless Population: A Review of the Literature. *Crisis,* 2009 (https://www.crisis.org.uk/media/20611/crisis_mental_ill_health_2009.pdf).

[9] Fazel S, Khosla V, Doll H, Geddes J. The prevalence of mental disorders among the homeless in western countries: systematic review and meta-regression analysis. PLoS Med 2008; 5: e225. 19053169 10.1371/journal.pmed.0050225 PMC2592351

[10] Nilsson S F, Hjorthoj CR, Erlangsen A, Nordentoft M. Suicide and unintentional injury mortality among homeless people: a Danish nationwide register-based cohort study. Eur J Public Health 2014; 24: 50–6. 23482619 10.1093/eurpub/ckt025

[11] Schreiter S, Bermpohl F, Krausz M, Leucht S, Rössler W, Schouler-Ocak M, The prevalence of mental illness in homeless people in Germany. Dtsch Aerzteblatt Online 2017; 114: 665–72. 10.3238/arztebl.2017.0665 PMC5963583 29070426

[12] Bickley H, Kapur N, Hunt IM, Robinson J, Meehan J, Parsons R, Suicide in the homeless within 12 months of contact with mental health services: A national clinical survey in the UK. Soc Psychiatry Psychiatr Epidemiol 2006; 41: 686–91. 16779501 10.1007/s00127-006-0087-6

[13] Fitzpatrick S, Pawson H, Bramley G, Wilcox S, Watts B. The Homelessness Monitor: England 2017. Crisis, 2017.

[14] Govenment Statistical Service. Harmonisation of Definitions of Homelessness for UK Official Statistics: A Feasibility Report. Government Statistical Service, 2019.

[15] Appleby L, Shaw J, Sherratt J, Amos T, Robinson J, McDonnell R, Safety First: Five-year Report of the National Confidential Inquiry into Suicide and Homicide by People with Mental Illness. UK Department of Health, 2001.

[16] StataCorp. Stata Statistical Software. Statacorp Llc, 2017.

[17] Gentil L. Determinants of suicidal ideation and suicide attempt among former and currently homeless individuals. Soc Psychiatry Psychiatr Epidemiol [Epub ahead of print] 9 Sept 2020. Available from: 10.1007/s00127-020-01952-3. 32909051

[18] Cavanagh JTO, Carson AJ, Sharpe M, Lawrie SM. Psychological autopsy studies of suicide: a systematic review. Psychol Med 2003; 33: 395–405. 12701661 10.1017/s0033291702006943

[19] Ayano G, Tesfaw G, Shumet S. The prevalence of schizophrenia and other psychotic disorders among homeless people: a systematic review and meta-analysis. BMC Psychiatry 2019; 19: 370. 31775786 10.1186/s12888-019-2361-7 PMC6880407

[20] Qin P, Agerbo E, Mortensen PB. Suicide Risk in Relation to Socioeconomic, Demographic, Psychiatric, and Familial Factors: A National Register–Based Study of All Suicides in Denmark, 1981–1997. Am J Psychiatry 2003; 160: 765–72. 12668367 10.1176/appi.ajp.160.4.765

[21] National Confidential Inquiry into Suicide and Safety in Mental Health. Annual Report: England, Northern Ireland, Scotland and Wales. University of Manchester, 2019.

[22] Hunt IM, Windfuhr K, Swinson N, Shaw J, Appleby L, Kapur N. Suicide amongst psychiatric in-patients who abscond from the ward: a national clinical survey. BMC Psychiatry 2010; 10: 14. 20128891 10.1186/1471-244X-10-14 PMC2845552

[23] Taylor H. Being Homeless And Experiencing Mental Health-Related Difficulties: Listening To And Learning From The Experiences Of Service Users Of A Designated Homeless Psychology Service. PhD, Leicester University, 2012.

[24] Appleby L, Shaw J, Amos T, McDonnell R, Harris C, McCann K, Suicide within 12 months of contact with mental health services: national clinical survey. BMJ 1999; 318: 1235–9. 10231250 10.1136/bmj.318.7193.1235 PMC27859

[25] Folsom DP, Hawthorne W, Lindamer L, Gilmer T, Bailey A, Golshan S, Prevalence and risk factors for homelessness and utilization of mental health services among 10 340 patients with serious mental illness in a large public mental health system. Am J Psychiatry 2005; 162: 370–6. 15677603 10.1176/appi.ajp.162.2.370

[26] Keats H, Cockersell P, Johnson R, Maguire N. *Psychologically Informed Services for Homeless People. Good Practice Guide*. Communities and Local Government, 2012 (https://www.pathway.org.uk/wp-content/uploads/2013/05/Psychologically-informed-services-for-Homeless-People.pdf).

[27] Faculty for Homeless and Inclusion Health. Homeless and Inclusion Health standards for commissioners and service providers. Pathway, 2018.

[28] Doyle E, Hamlet N. Homeless and Inclusion Health. NHS Health Scotland, 2019.

[29] UK Drug Policy Commission. A Fresh Approach to Drugs. UK Drug Policy Commission, 2012.

