# Supplementary material for: Suicide by homeless patients in England and Wales: national clinical survey
Source: BJPsych Open. 2021 Mar 12;7(2):e65. doi: 10.1192/bjo.2021.2 (PMC8058935; doi:10.1192/bjo.2021.2)
Supplement: Supplementary file 1 [file S2056472421000028sup001.docx]

Supplementary Appendix 1

Logistic regression analysis to explore potential confounding effects was carried out in forwards stepwise fashion. Missing data was imputed via multiple imputations with fully conditional specification (van Buuren & Groothuis-Oudshoorn, 2011), with each variable having its own imputation model. All analyses were carried out via RStudio 3.4.3 (RStudio Team, 2016), namely, the built-in stats package for logistic regression and mice package for interpolation of missing values (van Buuren & Groothuis-Oudshoorn, 2011). Model A has demographic and clinical characteristics as well as method of suicide entered as predictors in one step. Models B, C, and D contain age, sex, and calendar year of death, respectively, as predictors to explore their potential confounding effects (see Table 3).

Even though model fit improved by adding age, sex, and calendar year (AIC(A)= 3907.84, AIC(B)= 3865.43, AIC(C)= 3848.31, AIC(D)= 3764.65), overall significance of predictors from model A did not change.

References:

RStudio Team. (2016). RStudio: Integrated Development for R. (3.4.3.) [Computer software]. RStudio, Inc. <http://www.rstudio.com/>

van Buuren, S., & Groothuis-Oudshoorn, K. (2011). mice: Multivariate Imputation by Chained Equations in R. Journal of Statistical Software, 45(3). https://doi.org/10.18637/jss.v045.i03

| **Supplementary Table 1.** Results of forwards stepwise logistic regression with homelessness as criterion variable | | | | | | | | | |
| --- | --- | --- | --- | --- | --- | --- | --- | --- | --- |
|  | Model A | | Model B (A + age) | | Model C (B + sex) | | Model D (C+ year) | | |
|  | β-estimates | CI (95% confidence limits) | β-estimates | CI (95% confidence limits) | β-estimates | CI (95% confidence limits) | β-estimates | CI (95% confidence limits) |  |
| Intercept | -6.26 | -8.14, -4.39 | -5.10 | -7.18. -3.02 | -4.94 | -7.09, -2.85 | 18.27 | -525.38, 561.92 |  |
| Ethnic minority | -0.13 | -0.48, 0.23 | -0.24 | -0.60, 0.12 | -0.24 | -0.60, 0.12 | -0.23 | -0.61, 0.14 |  |
| Unemployed | 1.6** | 1.10, 2.10 | 1.43** | 0.93, 1.93 | 1.40** | 0.94, 1.86 | 1.37** | 0.87, 1.86 |  |
| Patient was a former member of the Armed Forces | -0.15 | -1.22, 0.93 | -0.09 | -1.18, 0.99 | -0.16 | -1.21, 0.89 | -0.24 | -1.17, 0.69 |  |
| Self-poisoning | -0.22 | -0.53, 0.08 | -0.20 | -0.51, 0.10 | -0.14 | -0.42, 0.13 | -0.14 | -0.40, 0.12 |  |
| In-patient at time of death | 0.91** | 0.43, 1.39 | 0.88** | 0.42, 1.35 | 0.91** | 0.46, 1.36 | 0.92** | 0.65, 1.18 |  |
| Patient discharged to housing, financial, employment problems | 2.90* | 0.39, 5.41 | 2.84* | 0.30, 5.39 | 2.81* | 0.25, 5.37 | 3.24* | 0.29, 6.18 |  |
| *p<0.05, **p<0.01 | | | | | | | | | |
